# The Wrong Place and Wrong Time for a Doctor to Get Ehrlichiosis

**DOI:** 10.7759/cureus.89472

**Published:** 2025-08-06

**Authors:** Justen M Aprile, Bettina Aprile

**Affiliations:** 1 Pediatrics, Penn State Health Milton S. Hershey Medical Center, Hershey, USA; 2 Family and Community Medicine, Penn State Health Milton S. Hershey Medical Center, Hershey, USA

**Keywords:** hepatitis workup, infectious causes of febrile jaundice, public health and safety, sepsis-induced thrombocytopenia, tick-borne infections, viral hepatitis

## Abstract

Febrile illnesses with associated laboratory abnormalities can have a wide differential diagnosis. While geographic location and seasonality can inform a workup, the overall incidence of specific conditions can lead to anchoring biases. Tick-borne illnesses are an important pathology to consider, despite known patterns and occurrences.Here, we discuss the case of a 42-year-old male pediatrician living in the central Pennsylvania area, with no significant past medical history, presenting with headache, diffuse myalgias, and several laboratory abnormalities, ultimately found to have ehrlichiosis in November, though not in an endemic region or the expected time of the year.

## Introduction

Tick-borne illnesses are often discussed in the differential diagnosis of a patient presenting with a fever of unclear etiology [1]. Season and geographic location determine the prevalence of various types of tick-borne illnesses. Central Pennsylvania is an endemic region for certain tick-borne illnesses, while others are far less likely to occur in this region [1,2]. We describe the case of a 42-year-old male pediatrician living in central Pennsylvania with tick exposure and a febrile, septic hepatitis.

## Case presentation

A 42-year-old male pediatrician living in the central Pennsylvania area with no significant past medical history presented with headache, diffuse myalgias, and several laboratory abnormalities. The patient was known to be in his usual state of health when he developed signs and symptoms consistent with a viral upper respiratory tract infection presumed to have been acquired from his children. Symptoms of cough, congestion, and minor myalgias, responsive to ibuprofen and acetaminophen, were present for three to five days before gradually subsiding. Just before complete symptom resolution, a large tick was removed from the patient’s right calf. Given the highly visible location of the attachment and the tick not found to be engorged, it was speculated that the tick was attached for not more than 12 hours. The entirety of the tick was removed, and symptoms neared resolution.

Approximately one week after tick removal, the patient abruptly developed more intense and diffuse myalgias, now only intermittently responsive to over-the-counter ibuprofen and acetaminophen. Headache became the most severe symptom and was located retro-orbitally and was bitemporal in nature. Symptoms progressed and intensified to the point where the headache was described as “the worst headache of my life.” The headache became so severe that the patient was unable to lie flat due to worsening of pain and pressure. The patient then noted progressively darkening urine, despite deliberate attempts to significantly increase hydration, extreme fatigue, and increased somnolence. Given the progression of symptoms and development of fever and rigors, the patient presented to the Emergency Department for further workup and evaluation.

Upon arrival at the Emergency Department, an extensive workup was undertaken. Significant laboratory abnormalities are detailed in Table 1. Blood and urine cultures were drawn, tick studies were obtained, and a prophylactic dose of doxycycline was administered, given tick exposure without any other obvious source. A liver ultrasound was obtained, which revealed hepatomegaly without other acute findings. The patient was subsequently admitted to the inpatient Family Medicine service for ongoing fever management, investigative workup, and pain control.

**Table 1:** Initial values obtained on admission.

Laboratory test	Patient value	Reference range
Alanine aminotransferase	215 U/L	0–41 U/L
Total bilirubin	3.0 mg/dL	0–1.2 mg/dL
Direct bilirubin	2.3 mg/dL	0–0.3 mg/dL
Alkaline phosphatase	222 U/L	40–130 U/L
Aspartate aminotransferase	176 U/L	0–40 U/L
Platelets	57 k/µL	150–350 k/µL
Lymphocytes	0.43 k/µL	1–3.4 k/µL
Procalcitonin	0.46 ng/mL	<0.08 ng/mL
C-reactive protein	15.89 mg/dL	<0.50 mg/dL

Given laboratory abnormalities, headache control was achieved through low-dose acetaminophen and PRN Dilaudid dosing. Laboratory abnormalities peaked two days after admission with alanine aminotransferase of 345 U/L, alkaline phosphatase of 224 U/L, and aspartate aminotransferase of 247 U/L. Clinically, headache, myalgias, and fever showed significant improvement following 24-48 hours of doxycycline therapy. The patient was subsequently discharged home with once-daily doxycycline and close PCP follow-up. At the time of discharge, antibody levels for Anaplasma were negative, Lyme was negative, and *Ehrlichia* antibodies were equivocal at 1:125 (IgM antibodies negative at <1:16). Serologies were rechecked approximately one month following discharge and were notable for *Ehrlichia chaffeensis* IgG antibodies with a significant increase to 1:512.

## Discussion

Human ehrlichiosis is a tick-borne infection caused by bacteria in the *Ehrlichia* genus, such as *Ehrlichia chaffeensis* and *Ehrlichia ewingii* [1]. Primary transmission is via the tick vector, the Lone Star tick, commonly among the white-tailed deer population [2].

The initial reported case of human monocytic ehrlichiosis was documented in 1991 [2]. The clinical presentation of ehrlichiosis is often characterized by the constellation of symptoms, including fever, chills, headache, malaise, myalgia, and nausea [1]. A rash is present in fewer than 30% of adults found to have the disease [1,3]. The rash, if present, is most typically maculopapular, petechiae, or diffuse erythema, most commonly on the extremities [3]. Symptoms most typically occur one to two weeks after exposure to the offending tick bite [1]. If illness becomes severe, symptoms in adults may include cough, diarrhea, confusion, and lymphadenopathy, and if left untreated, progression can occur to acute respiratory distress syndrome and disseminated intravascular coagulation, enteric nervous system involvement, and renal failure [1]. Abnormal laboratory findings that are most characteristic of ehrlichiosis infection include leukopenia, thrombocytopenia, and elevations in hepatic aminotransferase levels [1]. Patients with human ehrlichiosis caused by *E. chaffeensis* require hospitalization in more than 50% of cases, and death can occur as early as the second week of illness, reported in up to 3% of cases [1].

The differential diagnosis for human ehrlichiosis is broad and includes many of the etiologies one might consider for any febrile illness, as highlighted in Table 2 [3].

**Table 2:** Differential diagnosis of febrile illness. Adopted from Snowden and Simonsen [3].

Illness	Example
Tick-borne diseases:	*Rickettsia rickettsii*, *Babesia microti*, *Anaplasma*, *Borrelia burgdorferi*
Viral pathology:	Epstein-Barr virus, cytomegalovirus, influenza, dengue, hepatitis, sepsis
Parasitic:	Malaria
Bacterial:	Sepsis (*Staphylococcus*, *Streptococcus*, *Neisseria*), leptospirosis, toxic shock syndrome, typhoid fever
Malignancies	
Immunologic:	System lupus erythematosus, hemophagocytic lymphohistiocytosis
Kawasaki disease	

National reporting systems use the following definitions for case criteria, needing to meet both the clinical and laboratory criteria for reporting. Clinical presentation with fever and one or more of the following: headache, myalgia, anemia, leukopenia, thrombocytopenia, or any hepatic transaminase elevation (our patient had fever, headache, myalgia, thrombocytopenia, and transaminase elevation) [1]. Additionally, the case must demonstrate the following laboratory criteria: a four-fold change in IgG-specific antibody titer against *E. chaffeenisis* antigen by immunofluorescence assay between paired serum samples (one in first week of illness and second two to four weeks later), or *E. chaffeensis* DNA in polymerase chain reaction assay (not collected in this case), *E. chaffeensis* antigen in biopsy or autopsy sample by immunohistochemical methods or isolation in cell culture [1]. Reported to the Centers for Disease Control and Prevention (CDC) via surveillance of symptoms (in our case, the initial IgG was equivocal at the time of admission, with a titer of 1:512 (four-fold increase) one month later).

*E. chaffeensis* ehrlichiosis has experienced an increased incidence since it first became a nationally notifiable disease in 1999. Incidence of E. chaffeensis was 0.8 cases per million in the year 2000 [1]. By 2008-2012, the incidence of *E. chaffeensis* ehrlichiosis had risen to 3.2 cases per million per year [1]. With an incidence of 0 to <0.3 cases per one million individuals in Pennsylvania, ehrlichiosis requires a large index of suspicion to diagnose and ultimately treat [1,2].

This differs significantly from the incidence of Lyme disease, the most prevalent and frequently reported tick-borne illness in Pennsylvania. *Borrelia burgdorferi*, spread by the common deer tick (*Ixodes scapularis*), can have an incidence of 0.1 to 50 cases per 100,000 population, and even higher ranges outside of the central Pennsylvania region [4].

Treatment of human ehrlichiosis, as recommended by the CDC, is doxycycline, which should be initiated for suspected cases, even prior to laboratory confirmed testing [1]. When treatment with doxycycline is initiated within the first five days of illness, improved outcomes are reported [1,3]. Dosing is typically 100 mg twice daily for 7-10 days, depending on illness severity, and can be continued for an additional three days after fever resolution [3]. Most typically, fever resolution occurs within 72 hours of doxycycline initiation [3].

Certain populations are at higher risk for more severe illness. Older patients, immunocompromised individuals, or those previously treated with sulfonamide antibiotics may experience more severe symptoms or complications [3]. Prompt recognition and intervention improve the likelihood of favorable outcomes [3].

## Conclusions

Our case demonstrates a patient whose febrile illness was a result of a tick-borne illness outside of an area of expected high incidence and during an unexpected season. This illustrates the need to consider, maintain, revisit, and revise a broad differential for febrile illness regardless of the region or timing. Early use of antibiotics such as doxycycline can be lifesaving during such workup. Relying solely on geographic or seasonal norms can result in a degree of anchoring bias.
